# Novel biallelic loss-of-function variants in *CEP290* cause Joubert syndrome in two siblings

**DOI:** 10.1186/s40246-020-00274-4

**Published:** 2020-06-29

**Authors:** Xiang Wang, Zhu Zhang, Xueguang Zhang, Ying Shen, Hongqian Liu

**Affiliations:** 1 Department of Obstetrics/Gynecology, Joint Laboratory of Reproductive Medicine (SCU-CUHK), Key Laboratory of Obstetric, Gynecologic and Pediatric Diseases and Birth Defects of Ministry of Education, West China Second University Hospital, Sichuan University, Chengdu, 610041 China; 2 Department of Obstetrics and Gynecology, West China Second University Hospital, Sichuan University, Chengdu, 610041 China; 3 Key Laboratory of Birth Defects and Related Diseases of Women and Children, Ministry of Education, Sichuan University, Chengdu, 610041 China

**Keywords:** Joubert syndrome, Copy number variation, Compound heterozygous variants, WES, CGH

## Abstract

**Background:**

Joubert syndrome (JS) is a rare genetic disorder, which can be defined by brain stem malformation, cerebellar vermis hypoplasia, and consequent “molar tooth sign” (MTS). JS always shares variety of phenotypes in development defects. With the development of next-generation sequencing, dozens of causative genes have been identified to JS so far. Here, we investigated two male siblings with JS and uncovered a novel pathogenesis through combined methods.

**Results:**

The siblings shared similar features of nystagmus, disorders of intellectual development, typical MTS, and abnormal morphology in fourth ventricle. Whole-exome sequencing (WES) and chromosome comparative genomic hybridization (CGH) were then performed on the proband. Strikingly, a maternal inherited nonsense variant (NM_025114.3: c.5953G>T [p.E1985*]) in *CEP290* gene and a paternal inherited deletion in 12q21.32 including exons 1 to 10 of *CEP290* gene were identified in the two affected siblings. We further confirmed the two variants by in vitro experiments: quantitative PCR and PCR sequencing.

**Conclusions:**

In this study, we first reported a novel causative mechanism of Joubert syndrome: a copy number variation (CNV) combined with a single-nucleotide variant in *CEP290* gene, which can be helpful in the genetic diagnosis of this disease.

## Background

Joubert syndrome (JS) is a genetic ciliopathy disorder with multiple neurologic features of brain malformations, hypotonia, ataxia, and intellectual disability first described in 1969 [1, 2]. With the medical use of magnetic resonance imaging (MRI), JS diagnostic criteria were importantly revised with “molar tooth sign” (MTS), an abnormally deep interpeduncular fossa in midbrain [3–5]. Sharing MTS has been also used to group any individual displaying additional non-neurological features to Joubert syndrome-related disorders (JSRD) [4]. To date, more than 30 gene variants have been identified in JS, and which allow to group JS into different subtypes (JBTS) [6–8]. Most of these gene variants exhibit autosomal recessive inheritance, and the top three are *C5ORF42*, *CC2D2A*, and *CEP290* in turn [9, 10].

As with other genes associated with JS, *CEP290* is a primary cilium-related gene encoding centrosomal protein 290, a large essential protein expressed in almost all tissues and playing a critical role in cell motility and division through effects on centrosome and cilia development [11–13]. Disrupted CEP290 protein (also known as NPHP6) has been found to loss function binding to cellular membranes and microtubules [14], and proved to activate ATF4, a transcription factor associated with cAMP-dependent renal disease in JS patients [15]. Up to now, dozens of variants in *CEP290* have been identified to cause JBTS5 (OMIM#610188) [16], a subtype of JS mainly associated with serve retinal and renal involvement in affected individuals [17]. However, almost all variants resulted in premature stop codon in *CEP290* mRNA and truncated, nonfunctional CEP290 protein due to nonsense or frameshift variants, and the most frequent in which was c.5668G>T [p.G1890*] [15, 18, 19]. Interestingly, only one research reported a deletion from intron 43 of *CEP290* to C12orf29 (chromosome 12 open reading frame 19) [20]; there were no other CNVs in *CEP290* were reported in JS patients yet.

In this study, we presented clinical and molecular findings from a family with two JS affected siblings. Both of them carried a novel nonsense variant and a novel deletion in *CEP290* inherited from their parents respectively. The pathogenesis is compound heterozygous variants lead to biallelic loss-of-function of *CEP290*, which has been associated with JS clearly.

## Results

### Clinical summary

**Fig. 1:**
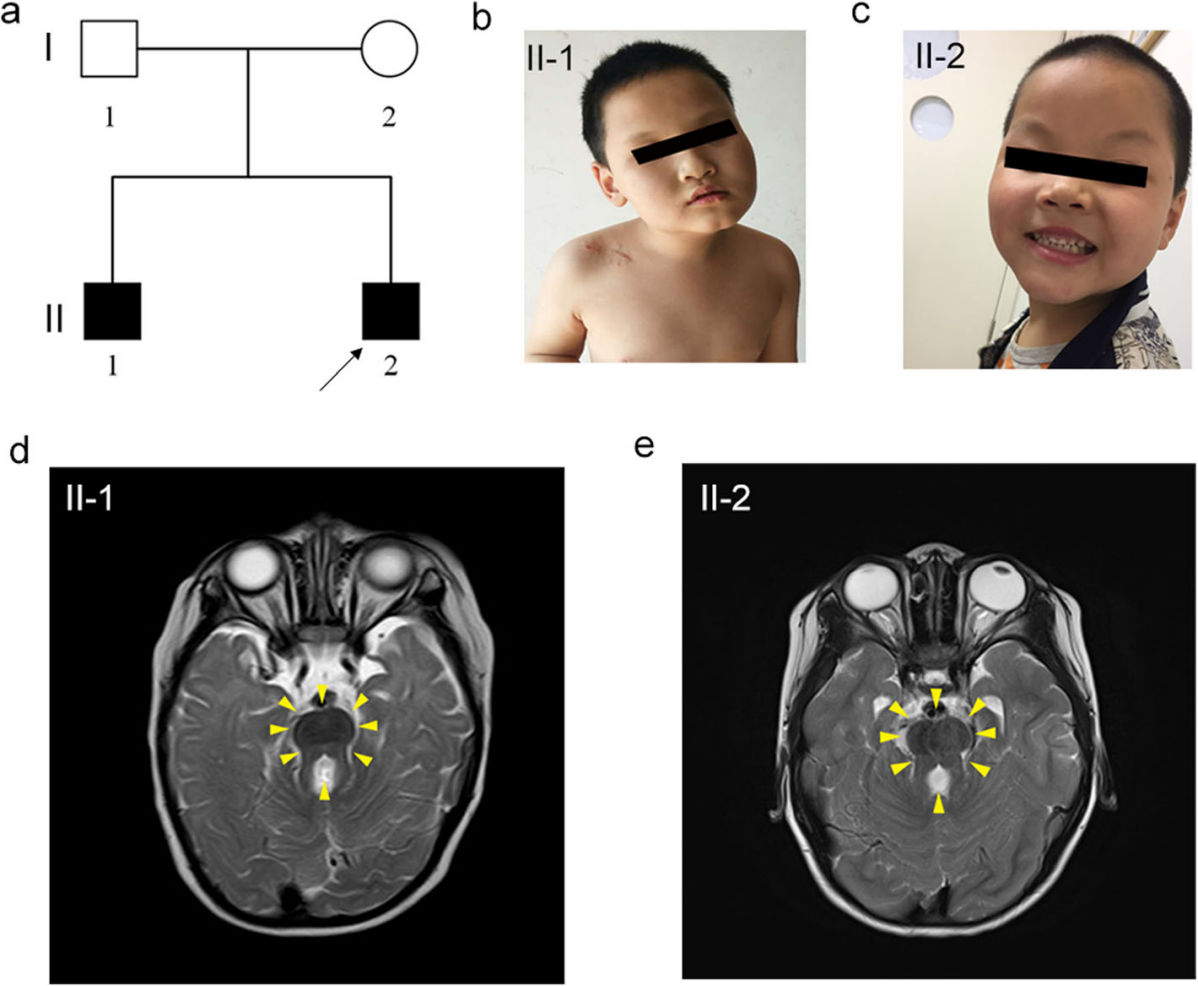
Clinical summary for a JS family. **a** Family pedigree. An unrelated natural couple who gave birth to two affected siblings (black arrow denotes the proband). **b**–**e** The affected siblings and their axial brain MRI (Yellow arrowheads showed the thick and long superior cerebellar peduncles, forming the roots of the so-called “molar tooth sign”)

An unrelated natural couple brought a 4-year-old boy with delayed development to the outpatient for genetic counseling (Fig. 1a). The proband exhibited features of moderate vision impairment, disorders of intellectual development, biparietal narrowing, and ataxia mainly on abnormal oculomotor (nystagmus) (Fig. 1b). Remarkably, the proband had a 10-year-old brother sharing the similar phenotype on vision impairment, intellectual development and oculomotor, and specially presenting gait disturbance (gait-limb incoordination) and abnormal form of the vertebral bodies (Fig. 1c). The axial brain magnetic resonance imaging (MRI) was then performed on these two siblings. Strikingly, hypoplastic cerebellar vermis, abnormal fourth ventricle, elongated superior cerebellar peduncles, and so-called “molar tooth sign” (MTS) were found on both of the two affected individuals (Fig. 1d, e). There was no functional and morphological abnormality of their kidney, lung, and liver so far. Thus, we initially diagnosed these two siblings with JS depending on the specific clinical characteristics. There was no phenotypic abnormality discovered in their parents.

### Molecular genetic analysis

**Fig. 2:**
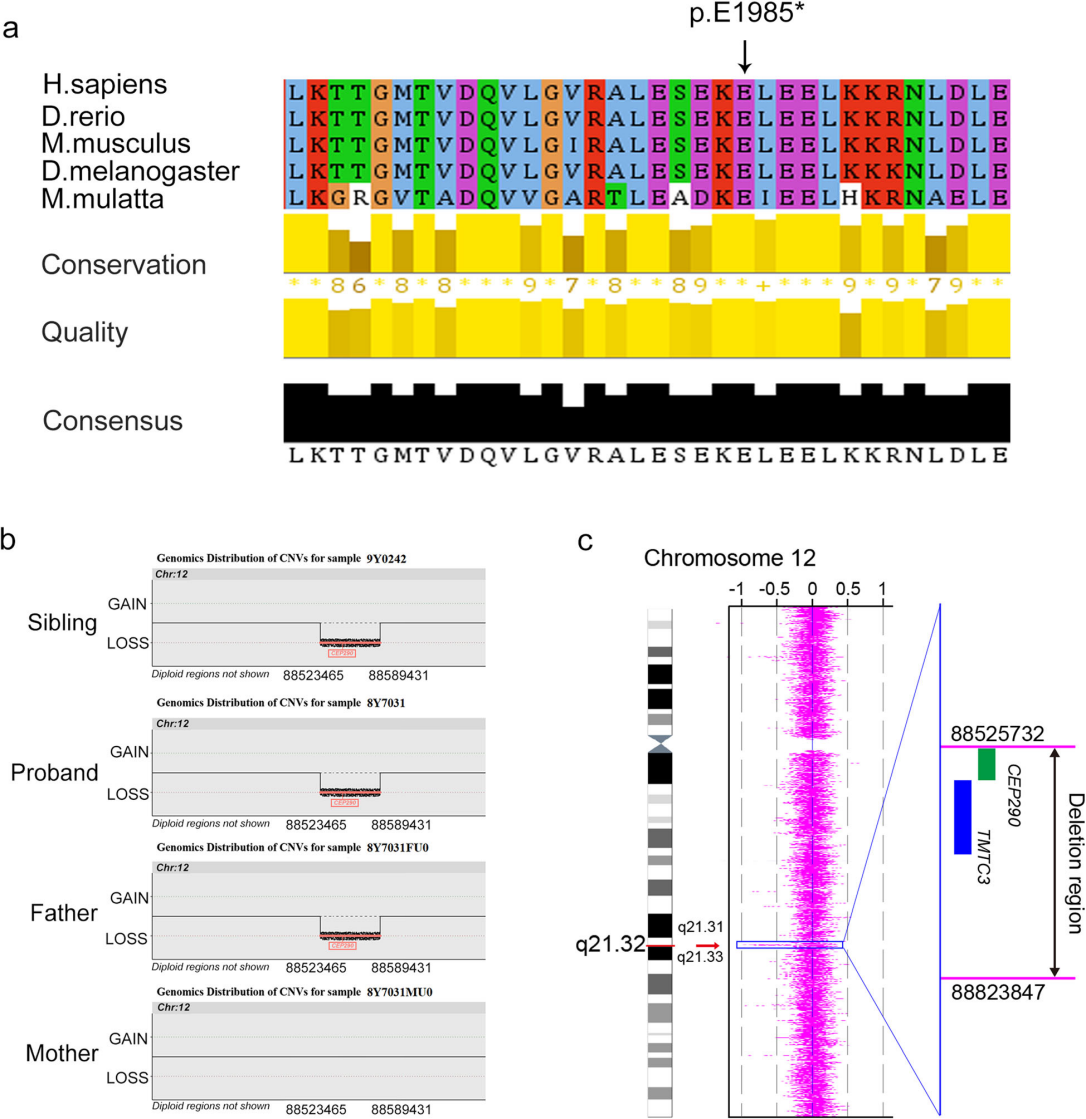
Variants identified from WES and chromosome CGH. **a** Multiple sequence alignment of the CEP290 protein for different species. (black arrow denotes the position of the variant) (c.5953G>T [p.E1985*]). **b** WES identified a suspected 65.97-kb deletion in 12q21.32 which affected *CEP290* in the father and siblings (8Y7031: proband, 9Y0242: sibling, 8Y7031FU0: father, 8Y7031MU0: mother). **c** Chromosome CGH confirmed a 298.1-kb deletion in 12q21.32 which affected *CEP290* in the family

To elucidate the underlying genetic cause of JS in this family, we performed WES on all the family members. Importantly, a novel heterozygous nonsense variant (c.5953G>T [p.E1985*]) in *CEP290* gene, a known causative gene of JS, was identified in the proband, his affected brother, and his mother but not the father. Furthermore, there was no report about this variation in any database, including ExAC browser, 1000 genome project, or GnomAD. In addition, this variant site is 100% highly conserved in many species (Fig. 2a). However, the nature of JS associated with *CEP290* is an autosomal recessive inheritance, so we considered that there should be another variant in *CEP290*. We further analyzed the WES data, and strikingly, a suspected 65.97-kb deletion in 12q21.32 (chr12, 88523465–88589431) was found in the two affected individuals and their father but absent in the mother (Fig. 2b), which covered exons 1 to 10 of *CEP290*. Furthermore, this *CEP290* deletion was confirmed on the proband by chromosome CGH analysis. As expected, a heterozygous 298.1-kb deletion in 12q21.32 (chr12, 88525732–88823847) was detected out, which gives rise to exon 1 to exon 6 deletion of *CEP290* (Fig. 2c). In view of the coding sequence (CDS) region of *CEP290* started in exon 2, the deletion detected in this family causes a complete translation deficiency. These findings concluded that the biallelic loss-of-function of *CEP290* variations was the genetic cause of JS in this family.

### The identification and negative effects of *CEP290* variants in the JS family

**Fig. 3:**
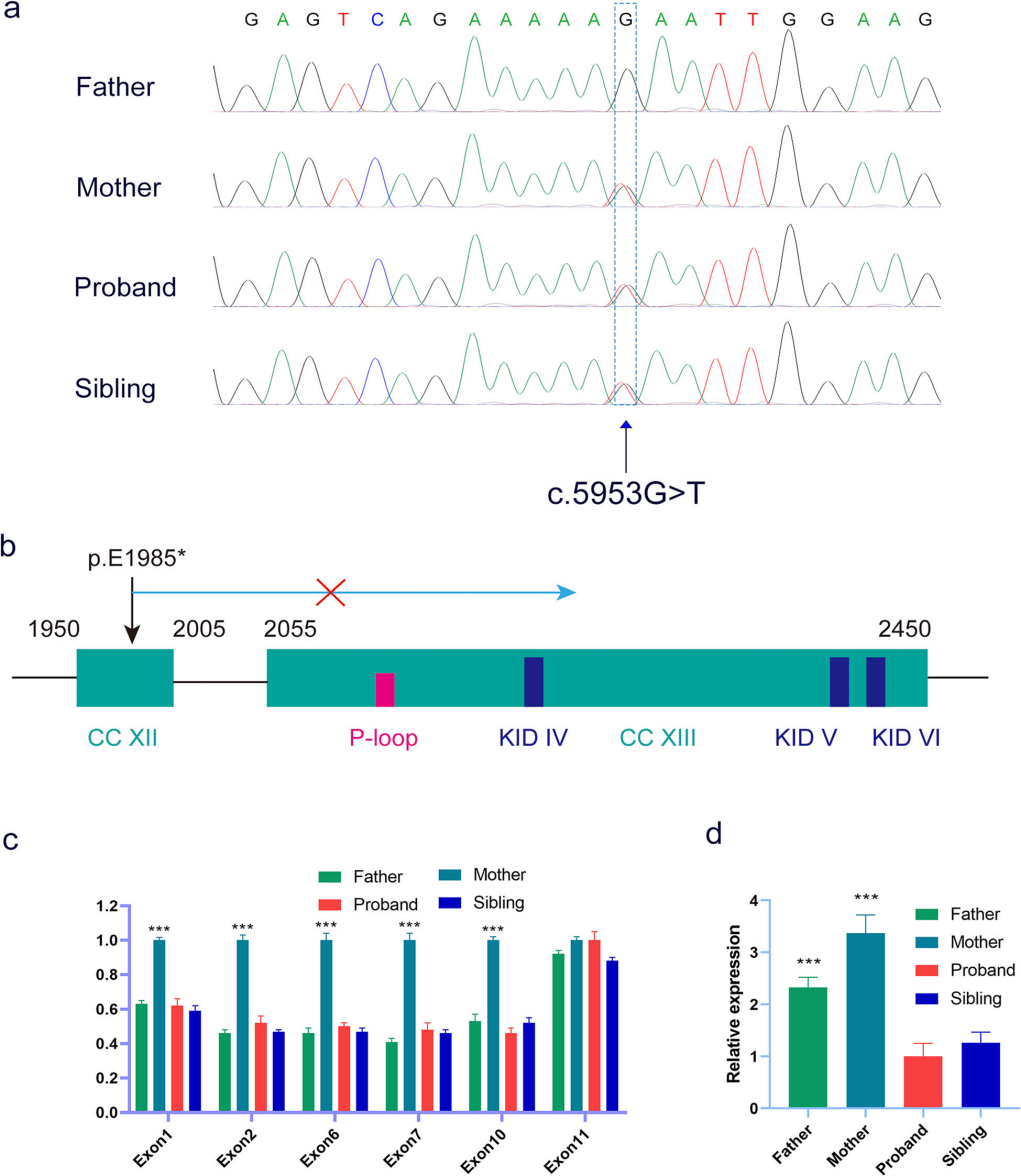
Verification and negative effect of the variants in *CEP290*. **a** PCR sequencing confirmed a c.5953G>T [p.E1985*] mutation in this family. **b** c.5953G>T [p.E1985*] in *CEP290* resulted in the loss-of-function of CEP290 protein due to lose numbers of functional domains. **c** Genomic qPCR revealed that father and the siblings only had relative half-fold copy for exon 1, exon 2, exon 6, exon 7, exon 10, and equivalent copy of exon 11 to mother. **d** qPCR showed significant decrease of *CEP290* expression in the two siblings compared to their parents. All the values are means ± SEM from three independent experiments, and statistical analysis was performed by one-way ANOVA. **P < 0.01, ***P < 0.001

To understand the putative contribution of the single-nucleotide variant (c.5953G>T [p.E1985*]) to the proband’s phenotype, we performed Sanger sequencing on this family, and this variant was observed in the proband, his affected brother, and their mother, meanwhile which was absent in their father (Fig. 3a). This variation resulted in a premature stop codon in transcribed mRNA (NM_025114.3, exon 43) and thus a truncated CEP290 protein, which leads to the missing of CC (coiled-coils) domain XIII, P-loop (ATP/GTP-binding site motif A) domain, KID (RepA/Rep+ protein kinase interaction domain) IV-VI(15) resulting in the loss-of-function of CEP290 protein (Fig. 3b).

To verify the CNV, qPCR was carried out to analyze genome relative copy number of exon 1, exon 2, exon 6, exon 7, exon 10, and exon 11 of *CEP290* in the family. Results showed that compared to the mother, the father and siblings only had relative half-fold copy for exon 1, exon 2, exon 6, exon 7, and exon 10, meanwhile exon 11 were equivalent (Fig. 3c). Furthermore, leukocyte mRNA levels of *CEP290* also revealed significant decrease in the two siblings compared to their parents by qPCR (Fig. 3d). Thus, these novel biallelic loss-of-function variants in *CEP290* could significantly reduce *CEP290* expression and further caused JS in these two siblings.

## Discussion

In this study, a novel genetic cause associated with a heterozygous nonsense variant combined a heterozygous deletion of a large fragment within *CEP290* was identified in two JS patients, which uncovered a novel pathogenesis of JS and provide more clue for genetic diagnosis of this disease

To date, variants of *CEP290* have been demonstrated with several genetic disorders more than JBST5, such as Leber congenital amaurosis (LCA10, OMIM#611755) [17], Meckel syndrome type 4 (MKS4, OMIM#611134) [21, 22], and Bardet-Biedl syndrome 14 (BBS14, OMIM#615991) [23, 24]. It is wondering that why variations in one gene could result in numbers of different syndromes. In 2010, Coppieters et al. reviewed over 100 distinct variants in *CEP290* and indicated that no clear genotype-phenotype correlation could be established and the reason may be insufficient understanding of second-site modifiers alleles, such as variants in other genes encoding ciliary protein and interact with *CEP290* [10]. In addition, we also hold the point that different variant subtypes in this gene may cause specific syndromes, as there are so many different domains with different vital functions in this large protein [14, 15]. In this study, we found two novel variants of *CEP290*, which may help to understand mechanisms of variation in *CEP290*.

Currently, WES has been frequently used and is an effective scheme for genetic disorder diagnosis, especially rare or non-fully clear diseases. However, some CNVs, including deletions and duplications, cannot be readily detected by WES [25]. Thus so, missing detecting CNV may give rise to unexplained genotype-phenotype uncorrelation in some disease. In our study, initially, in view of the proband shared the typical JBTS5 phenotype, but their parents claimed just a heterozygous *CEP290* nonsense variant was found in prior genetic diagnosis (details unknown). We therefore performed WES and chromosome CGH to focus on both SNV and CNV in *CEP290*. Expectedly, nonsense variant and deletion were detected out in the two patients. Our investigation of JS in this family showed it was very essential to use combined methods to study pathogenesis of genetic disorders.

## Conclusions

In conclusion, in this study, we unveiled two new variants, a SNV and a CNV of *CEP290*, which caused a complete translation deficiency of CEP290 leading to JS in two siblings. Specially, such deletion over 100 kb in *CEP290* detected in JS individuals was first reported. These findings may help to conclude and reveal specific variation types in *CEP290*, which is benefit for the diagnosis and then for the future precision treatment of JS.

## Methods

### Subjects

Peripheral blood samples were obtained from the proband and his family after informed consent was signed respectively. Experiment on human subjects was approved by the Ethical Review Board of West China Second University Hospital, Sichuan University. The two affected siblings’ brain MRI results were evaluated by two pediatric neuroradiologists.

### Whole-exome sequencing and chromosome comparative genomic hybridization

Genomic DNA was extracted from peripheral blood leukocytes using whole blood DNA purification kit (TIANGENE). For WES, exons were captured from 1 μg genomic DNA using Agilent SureSelect Human All Exon V6 Kit and then sequenced on Illumina HiSeq X system under instructions provided by the manufacturer. Functional annotation was performed through ANNOVAR and data were filtered by public database, such as ExAC, 1000 Genomes Project, and HGMD.

For chromosome comparative genomic hybridization, genomic DNA was digested with Nsp1 and ligated to adaptors. PCR amplicon was performed using primer pairs provided by the manufacturer, and the products were then purified and digested with DNase I to produce varying length DNA fragments. The fragments were labeled with biotinylated base and then hybridized with a pre-equilibrated Affymetrix chip Cytoscan HD chip. The chip consists of 2,670,000 probes that include 1,950,000 non-polymorphic probes for analysis CNVs. After washing and staining, the chip was scanned with the GeneChip Scanner and the signal intensity for each marker is assessed. The results were analyzed using the Chromosome Analysis Suite (version 3.3) with regard to aberrations minimally sizing 100 kb and according to public database including Database of Genomic Variants (DGV), Database of Chromosomal Imbalance and Phenotype in Humans using Ensembl Resources (DECIPHER), and UCSC human genome build 19. The analysis results were showed as log2 ratio of the intensities and allele peak distribution.

### Genomic DNA sequencing and quantitative PCR

Genomic DNA was obtained from peripheral blood leukocytes using whole blood DNA purification kit (TIANGENE). PCR amplification was performed using primer pairs (Table 1) designed to cover variant identified by WES. Sequencing of PCR products was conducted on an ABI377A DNA sequencer (Applied Biosystems). Data were evaluated using the Chromas software.

**Table 1:** Primers used in the current study

Primers	Forward	Reversed
CEP290 SNV	5′ TAAATTCCACAGAGCCGATAAA 3′	5′ ACAGCCCAAGAAATGAGGTT 3′
*CEP290* exon 1	5′ GTTCCACGCCTTCTCATCAT 3′	5′ TGCCAGGAGAGCCTACAGTT 3′
*CEP290* exon 2	5′ AGGTGGAGCACAGTGAAAGAA 3′	5′ TCTGCCAGTTCTTCTTGACG 3′
*CEP290* exon 6	5′ AGTCTGCAGGTGGACGAGAT 3′	5′ CCAACTCCTTTTCCATGTCC 3′
*CEP290* exon 7	5′ CAGCCTAGGCGACAAAGACT 3′	5′ CCTTTGTTGAACCACCACAA 3′
*CEP290* exon 10	5′ GGACACTTATGGCTGCGTTT 3′	5′ CATCAGTCATCTTCTCCATTTCC 3′
*CEP290* exon 11	5′ CATCAGTTTGCAACAACTCTTGA 3′	5′ TTTTGCATTGACAGCTACCAT 3′
*CEP290* mRNA	5′ AAAGTTGACCCAGATGACCT 3′	5′ AAACCGAGTATCTCGTCCAC 3′
Human GAPDH	5′ ATGTTCGTCATGGGTGTGAA 3′	5′ GTCTTCTGGGTGGCAGTGAT 3′

Total RNA was extracted using TRIzol reagent (Invitrogen, 15596026), and then reversed to cDNA through RT reagent Kit (Takara, RR037A). Triplicate quantitative PCR for genomic DNA or cDNA was performed using SYBR Green qPCR Master Mix (Bimake, B21202) on an iCycler RT-PCR Detection System (Bio-Rad Laboratories). Delta-delta CT value analysis method was used to evaluate genome exon 1, exon 2, exon 6, exon 7, exon 10, and exon 11 relative copy number, or exon 3 to 5 mRNA expression for all samples. Target and internal control gene primer pairs were listed below (Table 1).

## Data Availability

The data that support the findings of this study are available from the corresponding author upon reasonable request.

## Abbreviations

JS: Joubert Syndrome
MRI: Magnetic resonance imaging
MTS: Molar tooth sign
JSRD: Joubert syndrome-related disorders
CNV: Copy number variation
WES: Whole-exome sequencing
CGH: Comparative genomic hybridization
SNV: Single-nucleotide variants
